# P-609. Assessment of Parents’ Awareness and Attitudes towards Respiratory Syncytial Virus and its Prevention in the US

**DOI:** 10.1093/ofid/ofae631.807

**Published:** 2025-01-29

**Authors:** Yoonyoung Choi, Erwan Berjonneau, Brian DWYER, Bianca Chun, Tanaz Petigara, Xavier Guillaume

**Affiliations:** Merck, Philadelphia, Pennsylvania; Oracle, Vallauris, Provence-Alpes-Cote d'Azur, France; Oracle, Vallauris, Provence-Alpes-Cote d'Azur, France; Merck, Philadelphia, Pennsylvania; Merck & Co., Inc., Philadelphia, Pennsylvania; Oracle, Vallauris, Provence-Alpes-Cote d'Azur, France

## Abstract

**Background:**

To assess the awareness and attitudes of parents towards respiratory syncytial virus (RSV) infection in the United States.

**Methods:**

We developed an online, quantitative survey consisting of 34 questions, informed by a literature review, qualitative interviews, and cognitive tests. Between October 20th and November 25th, 2023, individuals who were expecting a child or had children aged ≤2 years were recruited through panels.

**Results:**

In total, 153 individuals were recruited, of which 73% were female and 57% had completed some schooling beyond high school. On average, they were 33.9 years old (standard deviation:7.2) and 78% had at least one child aged ≤2 years in their household, while 48% were expecting a child.

Most parents (88%) agreed that having their children immunized is a good way to protect them from diseases, and 84% agreed that they follow their healthcare provider’s recommendation for their children’s immunizations. However, 66% said they were concerned about serious side-effects of immunizations, and 54% question the value of some types of immunizations.

Level of knowledge about RSV varied among parents, with 35% reporting that they knew a lot about it, 39% that they were somewhat knowledgeable and 20% that they knew what it is but not much else. Very few (5%) had only heard of RSV but knew nothing more, and just 1% had never heard of RSV at all. Among parents who were aware of RSV (n=151), 63% were moderately or extremely concerned about RSV in children, with 33% extremely concerned. 34% of parents with children had at least one child who had ever been diagnosed with RSV, of which 60% visited urgent care or an emergency department and 46% were hospitalized.

About half of parents (46%) were aware of immunizations newly available for RSV. 90% strongly or moderately agreed that RSV prevention is important for children, and 78% would be eager to ask their physician about RSV immunization options.

**Conclusion:**

Findings show that most parents in the US are concerned about RSV in children and almost all think that RSV prevention is important for children. While a majority follow their provider’s recommendation, they also expressed concerns related to immunizations.

**Disclosures:**

**Yoonyoung Choi, PhD, MS, RPh**, Merck: Employee|Merck: Stocks/Bonds (Private Company) **Bianca Chun, MPH**, Merck Sharp & Dohme LCC, a subsidiary of Merck & Co., Inc., Rahway, NJ.: Employee **Tanaz Petigara, PhD**, Merck & Co., Inc.: Employee|Merck & Co., Inc.: Stocks/Bonds (Public Company)

